# Progestin-Primed Ovarian Stimulation Versus Mild Stimulation Protocol in Advanced Age Women With Diminished Ovarian Reserve Undergoing Their First *In Vitro* Fertilization Cycle: A Retrospective Cohort Study

**DOI:** 10.3389/fendo.2021.801026

**Published:** 2022-01-24

**Authors:** Xiaoyu Tu, Bingbing You, Miaomiao Jing, Chenxi Lin, Runju Zhang

**Affiliations:** Key Laboratory of Reproductive Genetics (Ministry of Education), Department of Reproductive Endocrinology, Women’s Hospital, Zhejiang University School of Medicine, Hangzhou, China

**Keywords:** progestin-primed ovarian stimulation, mild stimulation, advanced age, diminished ovarian reserve, cumulative clinical pregnancy rate, cumulative live birth rate

## Abstract

**Objective:**

To assess and compare the feasibility of progestin-primed ovarian stimulation (PPOS) protocol with mild stimulation protocol for advanced age women with diminished ovarian reserve (DOR) undergoing their first *in vitro* fertilization (IVF)/intracytoplasmic sperm injection (ICSI) cycle.

**Methods:**

Patients aged ≥35 years and DOR undergoing their first IVF/ICSI cycle were enrolled in this retrospective cohort study: 139 and 600 patients underwent the PPOS and mild stimulation protocols, respectively. The primary outcomes were cumulative clinical pregnancy rate (CCPR) and cumulative live birth rate (CLBR). The secondary outcomes were the number of oocytes retrieved and top-quality embryos.

**Results:**

There was nearly no significant difference of baseline characteristics between the two groups. Although a greater amount of total gonadotropin (1906.61 ± 631.04 IU vs. 997.72 ± 705.73 IU, *P*<0.001) and longer duration of stimulation (9 (10–7) vs. 6 (8–4), *P*<0.001) were observed in the PPOS group, the number of retrieved oocytes (3 (6–2) vs. 2 (4–1), *P*<0.001) and top-quality embryos (1 (2–0) vs. 1 (2–0), *P*=0.038) was greater in the PPOS group than the mild stimulation group. Meanwhile, the incidence of premature luteinizing hormone (LH) surge rate was significantly lower in the PPOS group (0.7% vs.8.3%, *P*=0.001) than the mild stimulation group. However, there was no significant difference in conservative CCPR, conservative CLBR, optimistic CCPR, and optimistic CLBR between the two groups (all *P*>0.05). A multivariate logistic regression model showed significant positive effects of the number of retrieved oocytes and number of top-quality embryos on conservative CCPR (OR=1.236, 95%CI: 1.048–1.456, *P*=0.012, OR=2.313, 95%CI: 1.676–3.194, *P*<0.001) and conservative CLBR (OR=1.250, 95%CI: 1.036–1.508, *P*=0.020, OR=2.634, 95%CI: 1.799–3.857, *P*<0.001) respectively, while significant negative effects of age were identified for conservative CCPR (OR=0.805, 95%CI: 0.739–0.877, *P*<0.001) and conservative CLBR (OR=0.797, 95%CI: 0.723–0.879, *P*<0.001).

**Conclusion:**

The PPOS protocol is an effective alternative to the mild stimulation protocol for advanced age patients with DOR, as it provides comparable reproductive outcomes and better control of premature LH surge. Further, more oocytes and top-quality embryos were obtained in the PPOS group, which had a positive association with conservative CCPR and CLBR.

## Introduction

With the successive implementation of China’s two-child policy and three-child policy and increasing delays in childbearing age, the number of advanced age women willing to have children is rapidly growing (1). However, there is a progressive decline in the number and quality of oocytes with age that results in diminished ovarian reserve (DOR) (2–4). In order to get pregnant, an increasing number of women with advanced age and DOR need to rely on assisted reproductive technology (ART). Unfortunately, these women are more prone to poor ovarian response (POR), a premature LH surge, and poor oocyte quality during ART treatment, leading to high cycle cancellation rate, low pregnancy and live birth rate, and high pregnancy loss rate (5). Choosing appropriate controlled ovarian stimulation (COS) protocols for these patients remains a great challenge for clinicians.

To date, there is no consensus on which strategy is the best choice for advanced age women with DOR (6). Conventional COS protocol usually uses high-dose gonadotropin for these patients during *in vitro* fertilization (IVF)/intracytoplasmic sperm injection (ICSI) cycles owing to POR and intense pituitary downregulation by gonadotropin-releasing hormone (GnRH) agonist or GnRH antagonist. Despite this, GnRH antagonist protocol is reportedly associated with about 0.34–8.0% failure to control premature LH surge in ovulatory women and the predominant risk factors include the increased age, DOR and POR (7). Meanwhile, high-dose gonadotropin is associated with a high risk of ovarian hyperstimulation syndrome, damage to oocyte quality, increased costs, and physical discomfort (4). Since many years, mild ovarian stimulation protocol has been used as a common alternative to conventional IVF protocol for these patients with the advantages of being safer, more patient-friendly and less expensive. Although fewer numbers of oocytes or embryos were obtained with mild stimulation, an updated systematic review showed that the live birth rates (LBR) and cumulative live birth rate (CLBR) were comparable to conventional stimulation in POR (8). Recently, the American Society for Reproductive Medicine (ASRM) also recommended that mild ovarian stimulation should be considered for IVF treatment in poor responders (9). However, mild stimulation IVF has been found to be associated with a higher cycle cancellation rate (CCR), predominantly owing to premature LH surge and lower response (10). Thus, continued exploration of appropriate COS protocols for advanced age women with DOR is an urgently needed.

The progestin-primed ovarian stimulation (PPOS) protocol, which was initially proposed by professor Kuang in 2015, involves pituitary suppression by oral progestins in the follicular phase until the ovulation trigger instead of GnRH analogues (11). A previous study showed that PPOS had a more robust control for preventing premature LH rise than GnRH antagonist in poor responders (7). A recent systematic review suggested similar ovarian response characteristics, pregnancy outcomes per transfer, and euploidy status of embryos with PPOS and GnRH analogues (12). However, whether PPOS can also be an alternative to conventional IVF treatment such as like mild stimulation for advanced age women with DOR remains unknown, because limited data were available to compare the two non-conventional protocols. Thus far, only one study showed a higher top-quality embryo rate, and a comparable clinical pregnancy rate was obtained in PPOS protocol versus mild stimulation among women aged >40 years. However, the sample size of the study was too small and many patients in the study had several previous IVF attempts before undergoing PPOS or mild stimulation cycle (1). Therefore, we conducted a retrospective cohort study with a larger sample size to assess the feasibility of PPOS protocol for advanced age women with DOR undergoing their first IVF/ICSI cycle by comparing it with the mild stimulation protocol.

## Methods

### Study Design

This retrospective cohort study was conducted in the Department of Reproductive Endocrinology of Women’s Hospital of Zhejiang University, School of Medicine. The study was approved by the Ethics Committee of the Women’s Hospital of Zhejiang University (reference: IRB-20210217-R). The data were anonymous, and the requirement for informed consent was therefore waived.

### Study Population and Data Collection

The characteristics and cycle parameters of infertility patients recorded in the database of our center were screened between January 2017 and December 2020. The inclusion criteria of patients were as follows: 1) age≥35 years; 2) DOR (AMH<0.5–1.1 ng/mL or AFC<5–7), 3) underwent their first IVF/ICSI procedure, and 4) use of either PPOS protocol or mild stimulation protocol. The exclusion criteria included: 1) uterine malformation, intrauterine adhesion, or abnormal endometrium; 2) endometriosis stage III–IV, adenomyosis or history of ovary surgery; 3) endocrine disorders such as hyperprolactinemia; 4) tuberculosis or other systemic diseases; and 5) women who received preimplantation genetic test.

The demographic, clinical, and laboratory data of included patients were collected from the hospital database in this study. Variables mainly included age, body mass index (BMI), antral follicle count(AFC), anti-Mullerian hormone (AMH), basal sexual hormone levels, infertility duration, infertility factor (female, male, both, and unexplained), total gonadotropin doses, total gonadotropin days and gonadotropin starting doses, sexual hormone levels on trigger day, endometrial thickness, number of oocytes retrieved, number of normal fertilizations (two pronuclei), and number of top-quality embryos (grade I and II embryos) and so on.

### Controlled Ovarian Stimulation Protocols

Ovarian stimulation protocol was performed as follows. In the PPOS group, patients received 10 mg/day oral medroxyprogesterone acetate (Xian Ju, Zhejiang Xianju Pharmaceutical Co., Ltd.) or 20 mg/day dydrogesterone (DYG) (Duphaston, Abbott Biologicals B.V., Netherlands) and received daily ovarian stimulation by injection of gonadotropin (HMG, LoBode, Livzon Pharmaceutical Co., Ltd., China) at 150–300 IU/day from the 2nd or 3rd day of the menstrual cycle until the trigger day. In the mild stimulation group, either 50–100 mg/day oral clomiphene citrate (Fertilan, Codal Synto Ltd., Cyprus) or 2.5–5 mg/day letrozole (Fu Rui, Jiangsu Hengrui Pharmaceutical Co., Ltd.) was administered for five consecutive days from cycle days 2–3, after which gonadotropin of 0–225 IU/day was injected until the trigger day. For both groups, the dose of gonadotropin was adjusted according to the number and size of developing follicles on ultrasound as well as serum concentrations of sexual hormones. Once three follicles of ≥16 mm diameter, two follicles of ≥17 mm diameter, or one follicle of ≥18 mm diameter was observed, the final stage of oocyte maturation was induced by administering 0.1 mg triptorelin (Decapeptyl, Ferring Pharmaceuticals, Germany) or 250ug recombinant HCG (Ovidrel, Merck Serono, Germany) and/or 3000–10000 IU hCG (Lizhu Pharmaceutical Trading Co., China). Transvaginal ultrasound-guided oocyte retrieval was conducted 32–36 h later.

All follicles measuring >10 mm in diameter were aspirated. The oocytes were fertilized by IVF or ICSI depending on semen parameters (13). Embryos were examined for the number and regularity of blastomeres and the degree of embryonic fragmentation and graded according to Cummins’s criteria (14). For patients who had undergone mild stimulation protocol, if the endometrium was in good condition (thickness≥8 mm, acceptable morphology) and there was no contraindication for transfer, a fresh cycle transfer could be performed on the third or fifth day after oocyte retrieval. Alternatively, viable embryos were vitrified and frozen, and patients underwent elective frozen embryo transfer. For patients who underwent PPOS treatment, all embryos were frozen. In our department, up to four top-quality embryos (including grade I and II embryos) were chosen and frozen by vitrification on the third day after oocyte retrieval. Superfluous embryos were placed in extended culture until they reached the blastocyst stage. During this stage, only good-morphology blastocysts were frozen on day 5 or 6.

### Endometrium Preparation and FET

For frozen-thawed embryo transfer cycles (FET), hormone replacement treatment (HRT) cycle and natural cycle were adopted for endometrium preparation. Briefly, the HRT cycle is suitable for all types of patients, especially for patients with irregular menstrual cycles, anovulation, or thin endometrium (endometrial thickness ≤ 6 mm on the day of LH surge during ovarian stimulation). The natural FET cycle was used for women with regular menstrual cycles.

For the HRT cycle, patients started to receive 2–8mg/day oral estradiol valerate (Progynova, Bayer, Germany) from day 3 of the menstrual cycle. From day 12 onwards, endometrium growth was monitored by transvaginal ultrasound and serum hormone levels were measured. When endometrial thickness was ≥8 mm and the duration of estrogen application was ≥12 days, two of these progestogens, namely oral DYG (20 mg/day); vaginal progesterone gel (90mg/day, Crinone, Merck Serono, Germany); or progesterone injection (60mg/day, Xian Ju, Zhejiang Xianju Pharmaceutical Co., Ltd.), were applied. Embryo transfer was scheduled on day 4 or 6 thereafter. Luteal support was maintained until 8–10 weeks of gestation or negative β-hCG detection 2 weeks after transfer.

For the natural cycle, transvaginal ultrasound scanning from cycle days 10–12 onward was used to monitor follicular growth. When the diameter of the dominant follicle was >14 mm, serum hormone levels were measured. Oral DYG (20 mg/day) was usually administered on the day of ovulation, and the cleavage-stage embryos or blastocysts were respectively transferred 3 or 5 days after ovulation. Luteal support was administered as above.

### Outcomes

The primary outcomes of the study were conservative and optimistic estimates of CLBR and CCPR per oocyte retrieval cycle, which was defined as the probability of the first live birth or clinical pregnancy from one round of ovarian stimulation, including all fresh and frozen embryo transfers from that oocyte retrieval cycle. Multiple births in a single pregnancy was considered a single live birth. The conservative estimate assumed that the patients who dropped out would not achieve clinical pregnancy or live birth if they had continued, while the optimistic estimate was based on the assumption that dropouts would have had the same clinical pregnancy or live birth rates as those who continued (15).

The secondary measures included the number of oocytes and top-quality embryos retrieved, top-quality embryo rate, premature LH surge rate, canceled ART cycle rate FET clinical pregnancy rate, FET implantation rate, FET pregnancy loss rate, and FET live birth rate. The incidence of premature LH surge was defined as the serum LH>15 mIU/mL on the trigger day, with or without dominant follicle rupture and increased serum progesterone. Conception was defined as a positive serum value of HCG. Clinical pregnancy was defined as the presence of an intrauterine gestation sac with fetal heart activity during ultrasound examination at 7 weeks of gestation. Ongoing pregnancy was defined as an intrauterine pregnancy with fetal heart motion at 12 weeks of gestation, but the absence of labor by the end of our research period. Live birth was defined as the delivery of an infant after 28 weeks of gestation. Pregnancy loss was defined as the outcome of any pregnancy that does not result in at least one live birth. The denominator of clinical pregnancy rate, ongoing pregnancy rate, and LBR in FET cycles was defined as total FET cycles. The denominator of pregnancy loss rate was defined as the number of total conception cycles. The top-quality embryos rate was defined as the number of top-quality embryos divided by the number of all split embryos. The canceled ART cycle rate was defined as the number of patients who had no viable embryo to transfer divided by the number of oocyte retrieval cycles. The FET implantation rate was defined as the number of gestational sacs divided by the number of embryos transferred in FET cycle.

### Statistical Analysis

Statistical analyses were conducted using IBM SPSS Statistics 23.0 software (IBM Corporation, Armonk, NY, USA). The normality of distribution was tested using the Shapiro–Wilk test. Normally distributed and skewed continuous variables were expressed as mean values ± standard deviations and median (Q3–Q1) respectively. Continuous variables were compared *via* Student’s t-tests or Mann–Whitney U test if applicable. Count data were presented as numbers and percentages and assessed using Pearson’s chi-square test or Fisher’s exact test when appropriate. A multivariate logistic regression analysis was performed to quantify the effects of related factors on the CLBR and CCPR. The positive results of univariate logistic regression (**Supplementary Table 1**) and clinical preferences were used to select potential factors; these included the type of stimulation protocol, maternal age, BMI, AFC, duration of infertility, number of oocytes retrieved, number of normal fertilization, and number of top-quality embryos. The data were presented as odds ratios (ORs) and 95% confidence intervals (CIs). The differences were considered statistically significant when the *P*-value was <0.05.

## Results

A total of 739 patients in our center’s database met the inclusion criteria and were enrolled in the study. Of these, 600 patients underwent mild stimulation protocol and 139 patients underwent PPOS protocol. The demographics and baseline characteristics of these patients are shown in **Table 1**. The mean patient age was 39.45 ± 2.99 years in the PPOS group and 39.98 ± 3.34 years in the mild stimulation group. The median levels of AMH and AFC in the PPOS group and mild stimulation group were 0.78 ng/mL and 5 and 0.65 ng/mL and 4, respectively. These data fully reflected the characteristics of patients’ advanced age and DOR. However, no significant difference was found in the age, BMI, AFC, AMH, infertility duration, primary infertility as well as infertility factor between the two groups (all *P*>0.05). The basal endocrine profiles included follicle-stimulating hormone (FSH), luteinizing hormone (LH), estradiol (E_2_) and progestogen (P). The basal LH levels showed significantly higher in the PPOS group than in the mild stimulation group [4.99 (6.36–3.80) vs. 4.51 (5.93–3.28), *P*=0.049], while the basal P levels were lower [1.06 (1.40–0.73) vs. 1.40 (1.78–1.00), *P*<0.001]. No significant difference was identified for basal FSH and E_2_.

**Table 1:** Demographics and basal characteristics of patients in both groups.

Characteristic	Mild stimulation (n = 600)	PPOS (n = 139)	*P*-value
Age	39.98 ± 3.34	39.45 ± 2.99	0.082
BMI (kg/m^2^)	22.29 ± 3.00	22.03 ± 2.62	0.357
Basal-FSH (mIU/mL)	9.36 (12.12–7.21)	9.37 (12.73–6.64)	0.543
Basal-LH (mIU/mL)	4.51 (5.93–3.28)	4.99 (6.36–3.80)	0.049
Basal-Estradiol (pmol/L)	119.45 (156.48–79.83)	133.54 (169.70–93.97)	0.052
Basal-Progesterone (nmol/L)	1.40 (1.78–1.00)	1.06 (1.40–0.73)	<0.001
AFC	4 (5–3)	5 (6–3)	0.081
AMH (ng/mL)	0.65 (1.07–0.39)	0.78 (1.30–0.45)	0.217
Primary infertility (%)	124 (20.7%)	29 (20.9%)	0.959
Infertility duration (years)	3 (5–1)	3 (6–2)	0.271
Infertility factor (%)			0.477
Female:	509 (84.8%)	113 (81.3%)	
Male	11 (1.8%)	5 (3.6%)	
Both	70 (11.7%)	19 (13.7%)	
Unexplained	10 (1.7%)	2 (1.4%)	

The cycle characteristics of the two groups are shown in **Table 2**. All the patients completed oocyte retrieval, and in 731 patients (other than for seven patients in the mild stimulation group and one patient in the PPOS group), at least one oocyte was successfully harvested. The initial doses of gonadotropin (213.77 ± 42.65 vs. 156.82 ± 46.56, *P*<0.001) and total doses of gonadotropin (1906.61 ± 631.04 vs. 997.72 ± 705.73, *P*<0.001) consumed in the PPOS group were significantly more than that of the mild stimulation group. Meanwhile the duration of ovarian stimulation in the PPOS group was also longer than that in the mild stimulation group (9 (10–7) vs. 6 (8–4), *P*<0.001). Accordingly, the number of oocytes retrieved (3 (6–2) vs. 2 (4–1), *P*<0.001), normal fertilization (2 (3–1) vs. 1 (2–1), *P* < 0.001) and top-quality embryos (1 (2–0) vs. 1 (2–0), *P*=0.038) were all larger in the PPOS group than mild stimulation group. During COS treatment, 50 cases in the mild stimulation group and one case in the PPOS group experienced a premature LH surge (LH>15 mIU/mL). Fortunately, there was no case of unexpected ovulation before oocyte retrieval. The incidence of premature LH surge rate was significantly lower in the PPOS group compared to the mild stimulation group (0.7% vs.8.3%, *P*=0.001). In the analysis of sex hormones on the trigger day, the E_2_ levels in the PPOS group were significantly higher than that in the mild stimulation group (3694 (7069–2152) vs. 2580 (3963–1390), *P*<0.001). However, the P and LH levels of the PPOS group were significantly lower than that of the mild stimulation group (1.36 (2.10–0.94) vs.1.90 (2.78–1.25), *P <*0.001, 3.68 (5.74–2.53) vs. 6.57 (9.82–4.32), *P*<0.001). Additionally, there was no significant difference between the two groups when the fertilization rate (67.6% vs. 65.6%), two pronuclei rate (57.5% vs. 55.8%) and top-quality embryo rate (68.0% vs. 65.0%) were analyzed (*P*=0.393, 0.481, and 0.343, respectively).

**Table 2:** Characteristics of cycle parameters in both groups.

Variables	Mild stimulation (n = 600)	PPOS (n = 139)	*P*-value
Initial doses of gonadotropin (IU)	156.82 ± 46.56	213.77 ± 42.65	<0.001
Total doses of gonadotropin (IU)	997.72 ± 705.73	1906.61 ± 631.04	<0.001
Duration of ovarian stimulation (days)	6 (8–4)	9 (10–7)	<0.001
LH on trigger day (mIU/mL)	6.57 (9.82–4.32)	3.68 (5.74–2.53)	<0.001
Premature LH surge rate	50 (8.3%)	1 (0.7%)	0.001
Estradiol on trigger day (pmol/L)	2580 (3963–1390)	3694 (7069–2152)	<0.001
Progesterone on trigger day (nmol/L)	1.90 (2.78–1.25)	1.36 (2.10–0.94)	<0.001
Number of oocytes retrieved	2 (4–1)	3 (6–2)	<0.001
Number of normal fertilization	1 (2–1)	2 (3–1)	<0.001
Fertilization rate (%)	1123 (67.6%)	382 (65.6%)	0.393
Two pronuclei rate (%)	956 (57.5%)	325 (55.8%)	0.481
Number of top-quality embryos	1 (2–0)	1 (2–0)	0.038
Top-quality embryo rate (%)	603 (68.0%)	199 (65.0%)	0.343

Comparisons of the clinical outcomes between both groups are shown in **Tables 3** and **4**. Among the 739 patients who initiated 739 ovarian stimulation cycles, 223 patients in the mild stimulation group and 56 patients in the PPOS group had to cancel their ART cycles because no viable embryo was available for transfer. The rate of ART cycle cancellation was similar between the two groups (*P*=0.494). Moreover, 35 patients in the mild stimulation group and 12 patients in the PPOS group froze all the embryos and did not undergo FET by the end of the study. Thus, a total of 350 FET cycles (mild stimulation group, [n=261], PPOS group, [n=89]) and 140 fresh ET cycles from the mild stimulation group were included in the analysis. Eighty-two patients in the mild stimulation group achieved intrauterine pregnancy; among them, 25 were from fresh embryo transfer (ET) and 57 were from FET. Nineteen patients achieved intrauterine pregnancy after FET in the PPOS group. No ectopic pregnancy occurred in either group. First trimester abortion occurred in nine patients after ET and 13 patients after FET in the mild stimulation group, and in two patients after FET in the PPOS group. Second and third trimester abortions occurred in one patient after ET and four patients after FET in the mild stimulation group, and in two patients after FET in the PPOS group. Thus, 72 patients (22 patients from ET cycle, 50 patients from FET cycle) in the mild stimulation group and 19 patients in the PPOS group achieved clinical pregnancy. Finally, 55 patients (15 patients from ET cycle, 40 patients from FET cycle) in the mild stimulation group and 15 patients in the PPOS group had live births. Among them, 11 patients in the mild stimulation group (four patients from the ET cycle, seven patients from the FET cycle) and two patients in the PPOS group had premature delivery. Five patients in the mild stimulation group had twin pregnancy. For FET cycles, the endometrial thickness before transfer showed no significant difference (*P*=0.731). The implantation rate (20.1% vs. 17.9%), clinical pregnancy rate (19.2% vs. 21.3%), ongoing pregnancy rate (17.2% vs. 19.1%), pregnancy loss rate (46.7% vs. 34.8%), and live birth rate (15.3% vs. 16.9%) were comparable between the two groups (all *P*>0.05). After taking fresh ET into consideration, there was no significant difference in the pregnancy outcomes including conservative CCPR (12.0% vs. 13.7%, *P*=0.589); conservative CLBR (9.2% vs. 10.8%, *P*=0.556); optimistic CCPR (13.2% vs. 15.8%, *P*=0.411); and optimistic CLBR (10.0% vs. 12.2%, *P*=0.438).

**Table 3:** Clinical FET outcomes of both groups.

Variables	Mild stimulation	PPOS	*P*-value
Number of FET cycles	261	89	
FET endometrial thickness (mm)	9.66 ± 1.95	9.76 ± 1.98	0.731
FET Implantation rate– no. (%)	69/343 (20.1%)	20/112 (17.9%)	0.601
FET Clinical pregnancy rate -no. (%)	50/261 (19.2%)	19/89 (21.3%)	0.654
FET Ongoing pregnancy rate- no. (%)	45/261 (17.2%)	17/89 (19.1%)	0.691
FET Pregnancy loss rate–no. (%)	35/75 (46.7%)	8/23 (34.8%)	0.315
FET Live birth rate– no. (%)	40/261 (15.3%)	15/89 (16.9%)	0.732

**Table 4:** Cumulative clinical outcomes of both groups.

Variables	Mild stimulation	PPOS	*P*-value
Number of FET cycles	261	89	
Number of fresh ET cycles	140	0	
Number of patients without viable embryos	223	56	
Number of patients without ET or FET	35	12	
Intrauterine pregnancy	ET:25	/	
	FET:57	19	
Ectopic pregnancy	0	0	
First trimester abortion	ET:9	/	
	FET:13	2	
Second and third trimester abortion	ET:1	/	
	FET:4	2	
Clinical pregnancy	ET: 22	/	
	FET:50	19	
Live birth	ET: 15	/	
	FET:40	15	
Premature birth	ET: 4	/	
	FET:7	2	
Twin pregnancy	ET: 1	/	
	FET:4	0	
Canceled ART cycle rate	223/600 (37.2%)	56/139 (40.3%)	0.494
Conservative CCPR-no. (%)	72/600 (12.0%)	19/139 (13.7%)	0.589
Conservative CLBR-no. (%)	55/600 (9.2%)	15/139 (10.8%)	0.556
Optimistic CCPR-no. (%)	79/600 (13.2%)	22/139 (15.8%)	0.411
Optimistic CLBR-no. (%)	60/600 (10.0%)	17/139 (12.2%)	0.438

A multivariate logistic regression model was established (**Table 5**). The results showed that significant positive (favorable) effects of the number of oocytes retrieved and number of top-quality embryos on conservative CCPR (OR=1.236, 95%CI: 1.048–1.456, *P*=0.012; OR=2.313, 95%CI: 1.676–3.194, *P*<0.001); and conservative CLBR (OR=1.250, 95%CI: 1.036–1.508, *P*=0.020; OR=2.634, 95%CI: 1.799–3.857, *P*<0.001), respectively. However, significant negative (adverse) effects of age were identified on the conservative CCPR (OR=0.805, 95%CI: 0.739–0.877, *P*<0.001) and conservative CLBR (OR=0.797, 95%CI: 0.723–0.879, *P*<0.001). Type of stimulation protocol, BMI, AFC, duration of infertility, and number of normal fertilization were not significant factors associated with conservative CCPR and conservative CLBR (all *P*>0.05).

**Table 5:** A multivariate logistic regression analysis of conservative CCPR and conservative CLBR in patients.

	Conservative CCPR		Conservative CLBR	
	OR [95% CI]	*P*	OR [95% CI]	*P*
Mild stimulation protocol vs PPOS	1.180[0.629,2.215]	0.606	1.161[0.578,2.330]	0.675
Age	0.805[0.739,0.877]	<0.001	0.797[0.723,0.879]	<0.001
BMI (kg/m^2^)	1.010[0.923,1.106]	0.823	1.009[0.911,1.117]	0.863
AFC	0.959[0.875,1.052]	0.381	0.997[0.904,1.100]	0.953
Infertility duration (years)	0.966[0.901,1.036]	0.328	0.985[0.913,1.062]	0.687
Number of oocytes retrieved	1.236[1.048,1.456]	0.012	1.250[1.036,1.508]	0.020
Number of normal fertilization	1.101[0.800,1.514]	0.555	1.250[1.078,1.451]	0.902
Number of top-quality embryos	2.313[1.676,3.194]	<0.001	2.634[1.799,3.857]	<0.001

## Discussion

Successful stimulation of advanced age women with DOR is one of the most frustrating aspects of IVF, as most COS protocols proposed to improve IVF outcomes in these patients provide disappointing results (16, 17). Owing to technical improvements of embryo cryopreservation and ‘freeze all’ strategies, the PPOS protocol has been increasingly used in recent years, which may provide better hope for this kind of special population. To our knowledge, this is the first study to evaluate the feasibility of PPOS protocol in advanced age women with DOR who underwent their first IVF cycle by comparing it with the mild stimulation protocol. In the present study, the CCPR and CLBR of the PPOS group were similar to that of the mild stimulation group, so were other reproductive outcomes in FET cycles. Meanwhile, more oocytes were retrieved and top-quality embryos obtained, as well as better control of premature LH surge was achieved in the PPOS group, although a greater dose of gonadotropin and longer stimulation duration were needed than in the mild stimulation group. Further analysis showed conservative CCPR and CLBR were positively associated with the number of oocytes retrieved and top-quality embryos, and negatively associated with age. Thus, our study demonstrates that the PPOS protocol is an effective alternative to the mild stimulation protocol for advanced age patients with DOR.

In this study, the incidence of premature LH surge rate and LH levels on the trigger day were significantly lower in the PPOS group than in the mild stimulation group, which was similar to the results reported in Peng et al’s study (1). Another self-controlled study comparing PPOS protocol with clomiphene-primed ovarian stimulation in infertile women with DOR also found that PPOS significantly suppressed the LH surge (18). It is well known that multi-follicular growth by COS results in increased production of estrogen, which can lead to a sudden LH surge and spontaneous ovulation before oocyte retrieval (19, 20). In particular, women with DOR have a high risk of a premature LH surge despite the use of GnRH antagonists (21). However, Chen et al. in their randomized controlled trial showed that PPOS had a more robust effect at preventing premature LH surge than GnRH antagonist in poor responders (7). These studies confirmed the efficacy of PPOS protocol to block the LH surge even for women with DOR. This effect is likely because the mechanism to inhibit LH surge by progesterone is centered on the progesterone receptor and mainly acts on the hypothalamus (22), which is different from GnRH analogues that directly act on pituitary GnRH receptors to cause pituitary downregulation. Specifically, if progestin is administered during the early part of the cycle before estrogen priming, it can inhibit transmission of the estradiol-induced signal through the inter-neuronal systems that link the estradiol-receptive neurons with the GnRH neurons (12). Meanwhile, high concentrations of progesterone reduced the frequency of the GnRH pulse, which further inhibited the synthesis of LH and the occurrence of the LH surge (12, 23). Yet, there are still many unknown factors about the endogenous LH surge and the exact role of progesterone in its occurrence; hence, further exploration is needed.

Another advantage of PPOS over mild stimulation protocol is the retrieval of more oocytes, normal fertilization, and top-quality embryos. This superiority seemed to be a result of significantly longer stimulation duration and the use of more gonadotropins in the PPOS group. However, previous studies have shown that increased use of gonadotropins did not benefit patients with DOR, especially those with low AFC (16, 18, 24). Thus, it was more likely that the improved number and quality of oocytes retrieved may not only be attributed to increased use of gonadotropin in the PPOS treatment but also attributed to the PPOS protocol itself. On the one hand, early follicular progesterone exposure in the PPOS program can effectively control the premature LH surge, avoid spontaneous ovulation, and/or premature luteinization of the follicles that affects the quality of the oocytes, thereby contributing to increased oocyte production. On the other hand, as the substrate of estrogen, progesterone is conducive to the synthesis of estrogen and the development of follicles, further improving the quality of oocytes. Correspondingly, significantly higher E_2_ levels and lower P levels were measured on the trigger day of the PPOS group than the mild stimulation group. Animal experiment also showed that the addition of oral progestin increased ovarian sensitivity to gonadotropin stimulation and improved luteal function in the cat (25). Clinical research found that elevated progesterone levels on the day of ovulation trigger had a negative effect on top-quality embryo rate and reduced the formation rate of top-quality blastocysts (26, 27). These evidences all indicate that the PPOS regimen is beneficial to patients with DOR. Certainly, randomized controlled trials of different COS protocols with similar gonadotropin doses among these special group of patients requires further exploration.

Our results showed a better tendency of reproductive outcomes in the PPOS group than the mild stimulation group despite no statistical significance. CCPR and CLBR, which are the most meaningful and clinically relevant outcomes for an infertile patient (28), were reported as conservative and optimistic estimates in our study because some patients who had not yet undergone fresh ET or FET by the end of the study, although all-embryo cryopreservation was performed. Therefore, the actual CCPR may lie between the conservative and optimistic estimates (12.0%–13.2%), as the CLBR (9.2%–10.0%). However, the CLBR observed in our study differed from that of previous studies. Devesa et al. found that CLBR was 25.9% at 38–39 years, 16.4% at 40–41 years, 7% at 42–43 years and 1.2% at age 44 years onwards among women ≥38 years who underwent their first IVF/ICSI cycle with a long GnRH agonist or a flexible GnRH antagonist protocol (29). Yang reported the conservative estimates of CLBR per woman in 401 women with POR to be 31.9% after at least four IVF/ICSI cycles. Specifically, the CLBR was 48.0% for <35 years, 30.1% for 35–39 years, and 16.9% for ≥40 years (30). Wang’s study included 1,825 POR women undergoing different kinds of protocols including mild stimulation and PPOS protocols. Specifically, the conservative and optimistic estimates of the CCPR at the first IVF/ICSI cycle from Groups 1 to 4 (Group 1, ≤35 years; Group 2, 36–40 years; Group 3, 41–43 years; Group 4, ≥44 years) were 40.36%, 32.50%, 17.22%, and 4.71%, respectively. As for CLBR, the conservative and optimistic estimates from Group 1 to 4 were 29.95%, 19.21%, 7.19%, and 0.71%, respectively (15). We speculate that differences of CCPR and CLBR among these studies may be attributable to differences in the characteristics and sizes of the patient populations, IVF/ICSI cycle rank as well as the COS regimen in each of these studies. Furthermore, consistent with many other studies (15, 30–32), our study showed that age was inversely correlated with CCPR and CLBR. The main reason was that the quantity and quality of oocytes usually decrease with age (33); the mitochondrial biogenesis in oocytes and the surrounding granulosa cells are severely impeded with age; and deoxyribonucleic acid instability increases with advanced age (34). Moreover, the probability of aneuploid embryos increased with age (35). Consistent with several studies showing that CLBR significantly increased with the number of oocytes retrieved (28, 29, 36), our study found that conservative CCPR and CLBR were positively correlated with the number of oocytes retrieved and the number of top-quality embryos. However, although more oocytes and top-quality embryos were obtained in the PPOS group in our study, there was no difference in the CCPR and CLBR between the two groups. This was likely because only fresh and subsequent FET results of one IVF/ICSI cycle were analyzed in our study, but for these advanced age patients with DOR, multiple IVF/ICSI cycles are usually required to improve the chances of clinical pregnancy and live birth. Therefore, it was difficult to obtain statistical differences based on only one oocyte retrieval cycle. Correspondingly, our study suggested that COS protocols exhibited no significant association with the conservative CCPR and CLBR, which was in agreement with Wang and Devesa’s studies (15, 29).

Our study has several strengths. To our knowledge, this is the first study to compare PPOS protocol with mild stimulation protocol among patients with DOR aged≥ 35 years. Previous studies usually analyzed their results per cycle but not per patient, and all patients were included regardless of their IVF/ICSI cycle rank. However, our study, aiming to overcome such methodological shortcomings, included only women undergoing their first IVF/ICSI cycle; thus, the results more directly reflected the effects of PPOS and mild stimulation protocol on this unique population, and did not interfered with the potential impact on the ovarian response of multiple previous IVF attempts. The sample size was larger than previous similar studies comparing the two protocols. Our study reported CCPR and CLBR as primary reproductive outcomes, which provides a more long-term view of the chance of ART success among the special population undergoing non-conventional protocols. Furthermore, we made conservative or optimal estimates of CCPR and CLBR to treat patients who had remaining embryos without ET rather than excluding them, so we believe that our results are more suitable for clinical reference.

Our study also has some limitations. First, we calculated the sample size powers for primary and secondary outcomes and found that the powers for CCPR and CLBR were relatively low (**Supplementary Figure 1**). Thus, the nonsignificant results of CCPR and CLBR between the two groups might be caused by the limited sample size, especially for the PPOS group (n=139). This is an exploratory study to investigate the availability of new clinical practice of PPOS protocol; hence, the low power of statistical analysis needs to be overcome by future randomized controlled clinical trials. Meanwhile, the study also has some inherent selection and confounding bias on account of its retrospective, single-center design, which is difficult to avoid. Hence, our results need to be further evaluated in future randomized controlled clinical trials with a larger and more appropriate sample size as well as similar basic clinical parameters (such as gonadotropin doses). Second, mild ovarian stimulation for IVF involves multiple strategies using the following agents as monotherapy or in combination, namely clomiphene, aromatase inhibitors, low-dose exogenous gonadotropins, GnRH antagonists, and late follicular-phase hCG/LH (10). Our study only included clomiphene/letrozole + gonadotropins as a mild stimulation regimen. It is unknown whether our results can be extrapolated to other types of mild stimulation regimens. Furthermore, only women aged ≥35 years with DOR participated in our study; thus, we are currently unable to draw conclusions about other population groups.

## Conclusion

The PPOS protocol is an effective alternative to the mild stimulation protocol for advanced age patients with DOR, as it achieves comparable reproductive outcomes and better control of premature LH surge. Moreover, more oocytes and top-quality embryos were obtained in the PPOS group than the mild stimulation group, which had a positive association with conservative CCPR and CLBR.

## Data Availability Statement

The original contributions presented in the study are included in the article/**Supplementary Material**. Further inquiries can be directed to the corresponding author.

## Ethics Statement 

The studies involving human participants were reviewed and approved by the Ethics Committee of Women’s Hospital of Zhejiang University (reference: IRB-20210217-R). Written informed consent for participation was not required for this study in accordance with the national legislation and the institutional requirements.

## Author Contributions

XT was involved in the design of the study and performed data analysis, interpretation and manuscript drafting. BY contributed to data collection and manuscript drafting. MJ and CL were responsible for data collection and checking. RZ was involved in the design, supervised the data analysis, and revised the manuscript. All authors contributed to the article and approved the submitted version.

## Funding

This study was supported by the National Nature Science Foundation of China (No. 82001626), the key research and development plan of Zhejiang Province (No. 2021C03098).

## Conflict of Interest

The authors declare that the research was conducted in the absence of any commercial or financial relationships that could be construed as a potential conflict of interest.

## Publisher’s Note

All claims expressed in this article are solely those of the authors and do not necessarily represent those of their affiliated organizations, or those of the publisher, the editors and the reviewers. Any product that may be evaluated in this article, or claim that may be made by its manufacturer, is not guaranteed or endorsed by the publisher.
