# Supplementary material for: Progestin-Primed Ovarian Stimulation Versus Mild Stimulation Protocol in Advanced Age Women With Diminished Ovarian Reserve Undergoing Their First In Vitro Fertilization Cycle: A Retrospective Cohort Study
Source: Front Endocrinol (Lausanne). 2022 Jan 24;12:801026. doi: 10.3389/fendo.2021.801026 (PMC8818948; doi:10.3389/fendo.2021.801026)
Supplement: Supplementary file 1 [file DataSheet_1.docx]

# Supplementary Figure

**Supplementary figure 1** Powers for the four primary clinical outcomes

The post hoc power of data analysis was estimated using STATA 15.0. The powers for the four primary clinical outcomes including number of oocytes retrieved, number of top-quality embryos, CCPR and CLBR were 99.80%, 85.08%, 10.40% and 11.40%, respectively.
