# Supplementary material for: Progestin-Primed Ovarian Stimulation Versus Mild Stimulation Protocol in Advanced Age Women With Diminished Ovarian Reserve Undergoing Their First In Vitro Fertilization Cycle: A Retrospective Cohort Study
Source: Front Endocrinol (Lausanne). 2022 Jan 24;12:801026. doi: 10.3389/fendo.2021.801026 (PMC8818948; doi:10.3389/fendo.2021.801026)
Supplement: Supplementary file 2 [file Table_1.docx]

# Supplementary Table

**Supplementary table1** A univariate logistic regression analysis of conservative CCPR and conservative CLBR in patients

| Parameter | conservative cumulative clinical pregnancy rate | | conservative cumulative live birth rate | |
| --- | --- | --- | --- | --- |
|  | aOR [95% CI] | *P* | aOR [95% CI] | *P* |
| Mild stimulation protocol vs PPOS | 1.161[0.675,1.998] | 0.590 | 1.199[0.656,2.191] | 0.556 |
| Age | 0.809[0.746,0.876] | <0.001 | 0.796[0.725,0.873] | <0.001 |
| BMI | 0.972[0.905,1.043] | 0.429 | 0.968[0.894,1.048] | 0.419 |
| Basal-FSH | 1.016[0.971,1.063] | 0.492 | 1.001[0.949,1.055] | 0.979 |
| Basal-LH | 1.029[0.948,1.116] | 0.496 | 1.025[0.935,1.124] | 0.596 |
| Basal-Estradiol | 1.000[0.998,1.003] | 0.909 | 1.000[0.997,1.003] | 0.960 |
| Basal-Progesterone | 0.899[0.652,1.238] | 0.512 | 0.833[0.576,1.204] | 0.330 |
| AFC | 1.004[0.927,1.088] | 0.918 | 1.034[0.948,1.127] | 0.453 |
| AMH | 0.751[0.372,1.519] | 0.426 | 0.805[0.369,1.757] | 0.586 |
| Infertility duration | 0.965[0.911,1.022] | 0.222 | 0.977[0.918,1.040] | 0.470 |
| Total dose of gonadotropin (IU) | 1.000[1.000,1.000] | 0.233 | 1.000[1.000,1.000] | 0.645 |
| Duration of ovarian stimulation (days) | 1.031[0.964,1.101] | 0.374 | 1.006[0.935,1.083] | 0.873 |
| LH on trigger day (mIU/mL) | 0.970[0.926,1.017] | 0.206 | 0.977[0.929,1.028] | 0.371 |
| Estradiol on trigger day (pmol/L) | 1.000[1.000,1.000] | 0.066 | 1.000[1.000,1.000] | 0.072 |
| Progesterone on trigger day (nmol/L) | 0.950[0.801,1.126] | 0.555 | 1.002[0.835,1.204] | 0.980 |
| Number of oocytes retrieved | 1.113[1.041,1.191] | 0.027 | 1.165[1.081,1.251] | 0.034 |
| Number of normal fertilization | 1.347[1.202,1.510] | <0.001 | 1.324[1.173,1.496] | <0.001 |
| Number of top-quality embryos | 1.890[1.597,2.236] | <0.001 | 1.890[1.5813,2.261] | <0.001 |
